# Buildings Contemplated and in Progress

**Published:** 1914-04-11

**Authors:** 


### Buildings Contemplated and in Progress.

Aberayon.—Tenders are invited for the conversion of Aberayon Workhouse into a cottage hospital. Particulars may be obtained upon application to Mr. B. C. Jones, clerk to the Guardians, Aberayon.

Belfast.—The Guardians have decided to carry out a number of improvements in connection with the workhouse buildings, involving an expenditure of about £10,000. Some of these include the erection of a new cooking kitchen for the infirmary, with stoves, the laying of new water mains, and the erection of fire escapes. It is also proposed to provide new lavatory and sanitary accommodation at the school buildings, the female side of the house, the convalescent department, and the male infirm department.

Bourne.—The Rural District Council have resolved to obtain tenders for an isolation hospital. The cost is estimated at over £1,000.

Bromley.—The Guardians have decided to apply to the Local Government Board for sanction to borrow £1,500 for alterations and additions to the workhouse.

Burton-on-Trent.—The General Committee of the Burton Infirmary have adopted a scheme for the improvement of the building, at an estimated cost of £6,000.

Clutton.—The Guardians have decided to carry out alterations and improvements at the workhouse, at an estimated cost of £2,728.

Exminster.—The Asylum Committee have recommended the Devon County Council to build eighteen cottages for the asylum attendants, at a cost not exceeding £6,000.

Grimsby.—Tenders are invited for the erection of verandahs and iron staircases at the Grimsby and District Hospital. All information for submitting tenders to be obtained at the offices of Mr. H. C. Scaping, architect, Court Chambers, Grimsby.

Lochgilphead.—Tenders are invited for additions and alterations to the kitchen department of the Argyll and Bute District Lunatic Asylum, Lochgilphead. Specifications may be obtained of the medical superintendent at the asylum.

Oakdale.—The Mynyddislwyn Urban District Council have approved plans for a cottage hospital for the Model Village authorities.

Sheffield.—Alterations are to be carried out at the Jessop Hospital, at an estimated cost of £15,000.

Tooting.—The Metropolitan Asylums Board have approved a scheme for the extension of the Tooting Bec Asylum, at an estimated cost of from £151,500 to £165,854.

Wisbech.—Over £1,300 of the £2,000 required has been subscribed towards the cost of a new wing to the North Cambridgeshire Hospital at Wisbech.

